# PhosCalc: A tool for evaluating the sites of peptide phosphorylation from Mass Spectrometer data

**DOI:** 10.1186/1756-0500-1-30

**Published:** 2008-06-23

**Authors:** Daniel MacLean, Michael A Burrell, David J Studholme, Alexandra ME Jones

**Affiliations:** 1 The Sainsbury Laboratory, John Innes Centre, Norwich Research Park, Colney Lane, Norwich, NR4 7UH, UK

## Abstract

**Background:**

We have created a software implementation of a published and verified method for assigning probabilities to potential phosphorylation sites on peptides using mass spectrometric data. Our tool, named PhosCalc, determines the number of possible phosphorylation sites and calculates the theoretical masses for the b and y fragment ions of a user-provided peptide sequence. A corresponding user-provided mass spectrum is examined to determine which putative b and y ions have support in the spectrum and a probability score is calculated for each combination of phosphorylation sites.

**Findings:**

We test the implementation using spectra of phosphopeptides from bovine beta-casein and we compare the results from the implementation to those from manually curated and verified phosphopeptides from our own experiments. We find that the PhosCalc scores are capable of helping a user to identify phosphorylated sites and can remove a bottleneck in high throughput proteomics analyses.

**Conclusion:**

PhosCalc is available as a web-based interface for examining up to 100 peptides and as a downloadable tool for examining larger numbers of peptides. PhosCalc can be used to speed up identification of phosphorylation sites and can be easily integrated into data handling pipelines making it a very useful tool for those involved in phosphoproteomic research.

## Findings

### Challenges of detecting phosphorylated residues in mass spectrometer data

Phosphorylation is probably the most common of protein post translational modifications (PTMs), with 30% of eukaryotic proteins estimated to be modified this way [1]. Phosphorylation is essential to the cell by playing a central role in signal transduction cascades, regulation of protein activity and protein-protein interactions. Therefore, protein phosphorylation is one of the most intensely studied PTMs. Protein phosphorylation can be detected as a mass shift (+79.99 Da) in mass spectra, which corresponds to the addition of HPO_3 _to a peptide, generally at serine, threonine or tyrosine residues. In the mass spectrometer, peptides fragment in predictable ways and programs such as MASCOT [2] use algorithms to match predicted fragmentation patterns of peptides from sequence databases to that observed in MS spectra. While these programs allow for modification to peptides, they do not explicitly compare the evidence that may support localisation of a modification to a specific residue rather than a neighbouring position. Nor are they explicit when the data cannot distinguish between alternatives. As phosphorylation may occur at rather common amino acid residues, it is not unusual for a peptide to contain several possible sites. The evidence that allows one to discriminate between two possibilities can be as low as one or two peaks in a mass spectrum. Alternatively, if the potential sites are well separated on the peptide, there may be direct evidence in the form of several well identified peaks to support one site over another.

It is important to be explicit about the level of confidence a mass spectrum can provide for a particular phosphorylation site because this information has a large impact on subsequent laboratory work (for example, identifying targets for site-directed mutagenesis). However, it is hard to evaluate MS data and accurately judge the information provided by MS2 fragmentation spectra (MS2 spectra are spectra from the first fragmentation, MS3 spectra are selected from the MS2 fragmentation and so forth) without time-consuming manual examination by experienced personnel. The interpretation of mass spectra of phosphopeptides, particularly from ion trap instruments, is further complicated by the tendency of phosphopeptides to preferentially fragment at the labile phosphoester bond (with neutral loss of -98 Da; H_3_PO_4_) often accompanied by poor fragmentation along the peptide backbone. This problem can be addressed (in ion traps) by a further fragmentation event (MS3) on the neutral loss product ion produced in the MS2 fragmentation event.

### An algorithm to identify the phosphorylation site with best support in the spectrum

Recently an algorithm has been developed to provide further support of peptide identifications from MS2 spectra by comparison to MS3 [3]. The use of such an algorithm in an analysis pipeline allows automatic phosphorylation site identification or allows pre-selection of spectra for manual identification. The method was subsequently developed to validate the position of phosphorylation from similar data [4]. The Olsen-Mann algorithm uses the four most intense peaks per 100 *m/z *units in an MS2 or MS3 spectrum, determines the theoretical masses of b and y ions, and makes corrections for the masses of the ions appropriate to whether the peptide sequence and spectrum are derived from an MS2 or MS3 spectrum. By calculating all possible b and y ions and all combinations of phosphorylation site the algorithm is able to work on peptide sequences with any number of potential phosphorylation sites. The algorithm counts the matches of the four most intense peaks to each theoretical b and y ion. A match is called whenever a peak from the spectrum falls within a user-specified window of error around the theoretical ion mass; the mass accuracy of the mass spectrometer used to generate the spectrum file should determine the size of the appropriate window of error.

The probability of phosphorylation [3] at a site is given as

(1) $$p(phos)=\left(\begin{array}{c} n\\ k \end{array}\right)\cdot P^{k}\cdot {\left(\text{1}-P\right)}^{\left(n-k\right)}$$

where

n = the total number of possible b and y ions,

k = the number of successful matches

P = 0.04 (as 4 peaks are allowed per 100 Da [2])

The probability score is

(2) -10log(*p*(*phos*))

### PhosCalc: an implementation of the algorithm

The algorithm has gained popularity as the number of large scale projects increase and is incorporated into the open source program MSQuant [4] but, surprisingly, is not available as a stand-alone tool. Therefore, we have implemented an exact version of the leading method described and verified in [5] and used in other published studies that calculates a probability based score for each potential phosphorylation site. An existing implementation of the algorithm, Ascore [6], described in [7] is available but unlike PhosCalc is apparently tied to an underlying human protein database for a compulsory peptide identification step, removing its applicability to data derived from other organisms. PhosCalc allows the user to provide a peptide sequence from any source. Unlike Ascore, PhosCalc will analyse data from MS2 and MS3 spectra, not just MS2 spectra. PhosCalc also permits the user to vary the window of error for peak matching allowing for analysis of data from mass spectrometers of different mass accuracy.

**Figure 1:**
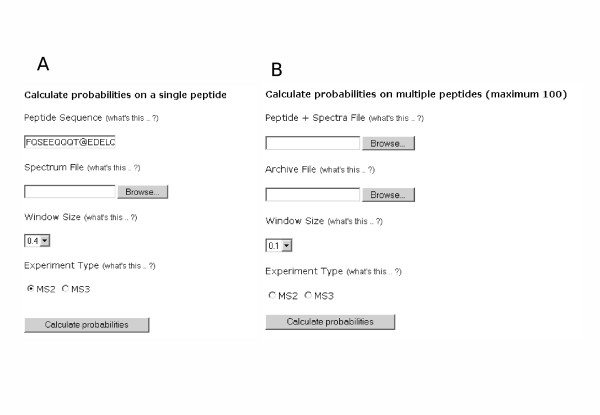
Data input screens from the web version of PhosCalc.

**Figure 2:**
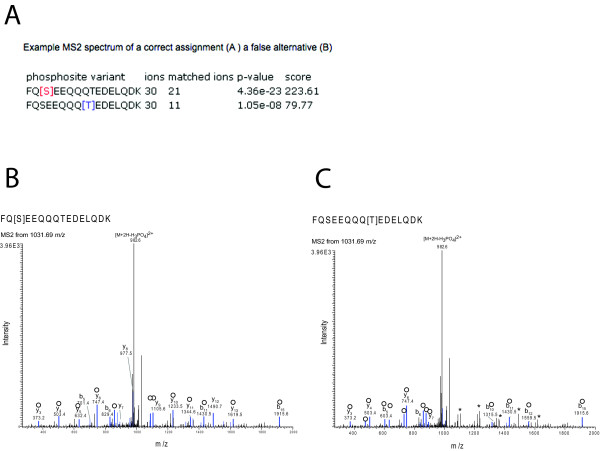
**Example of results of PhosCalc for the peptide FQSEEQQQTEDELQDK with annotated MS2 spectrum from Sequest**. A) PhosCalc output. B) Spectrum from a correct assignment. C) The same spectrum as 'B' with an incorrect assignment. Peaks marked in blue and marked with empty circles are matched to b or y ions by Sequest and * marks those peaks that do not match with the incorrect position of phosphorylation. For clarity only singly charged b and y ions without further loss of OH or NH_3 _groups are annotated.

Our implementation, called PhosCalc, is available as a web-tool or downloadable Perl script. In both versions, the user must provide predicted peptide sequences, the number of phosphorylation sites and a corresponding mass spectrum in the Sequest DTA format. Data can be entered via the simple web-interface for one to 100 peptide sequences (Figure 1A); two files are required: a list of peptide sequences and the zipped peak lists as dta files. The standalone package can handle an unlimited number of sequences. The PhosCalc tool returns an easily interpreted table showing probability of phosphorylation (defined as *p(phos) *in formula 1) and score (defined in formula 2) for each combination of phosphorylation sites and the number of peak matches in the spectrum for that variant (Figure 2B). Our implementation uses mono-isotopic residue masses and the value for cysteine is unmodified (i.e., not alkylated). These values can be easily altered in the downloadable version of PhosCalc. The variable oxidation of methionine is included by 'M*' with the addition of +15.99 Da. The mass value for phosphorylation is +79.9799 Da and is indicated by @ or # in the peptide sequence. As MS3 spectra are triggered by loss of phosphate plus water, the loss of 18.0105 Da is expected to replace phosphorylation site in MS3 spectra.

### Using PhosCalc

As a minimum, PhosCalc requires that the user provide a peptide sequence that is thought to be phosphorylated and a corresponding mass/intensity (dta) file from an MS experiment. File formats for both the command line and web versions are equivalent. The command line version runs non-interactively from a single command line invocation and creates output in a tab-delimited text file so that the tool can be easily incorporated into pipelines and workflows. The following description is of web-tool usage; instructions on how to run the downloadable version are available in the README file that comes with the download.

• On the main PhosCalc page [8] insert into the box Peptide Sequence (Figure 1A) the amino acid sequence of the phosphorylated peptide and the markers representing the potentially phosphorylated peptides. The amino acid sequence of the peptide may be represented using the following standard IUPAC amino acid symbols: A, C, D, E, F, G, H, I, K, L, M, N, P, Q, R, S, T, V, W or Y and the potentially phosphorylated sites may be identified by insertion of one of @, # or ^ (as far as PhosCalc is concerned these symbols are interchangeable, but some upstream analysis software make distinctions) after the putative phosphorylation site suggested by the MS software; e.g., the peptide sequence YNS#DTPEGVNSNWQR indicates that the peptide is thought to carry a single phosphorylation, possibly on the first serine (the calculator will assess and return the likelihood of phosphorylation at all possible sites within the peptide, irrespective of the putative phosphorylation site suggested by the MS software). An oxidised methionine residue may be indicated by entering 'M*'.

• Select the spectrum file to be evaluated (Figure 1A); this is the output from the MS machine that represents the mass peaks associated with this peptide. PhosCalc expects that this is to be in .dta format file. A dta file is a mass/intensity pair list that is a representation of the original MS/MS spectrum and consists of two columns of decimal numbers separated by one or more space characters and ended by a carriage return. Examples are provided on the web site and with the downloadable tool.

• Select a Window Size (Figure 1A). The hypothetical mass peaks derived from the peptide sequence are matched with the peaks from the mass spec by allowing a window of error. This option defines the width of this window of error.

• Select the Experiment Type (Figure 1A).

• Select whether the data came from an MS2 or MS3 experiment. If this is not selected, an MS2 experiment will be assumed. The effect of selecting MS3 is that the dehydration values will be used for #, @ and ^ symbols (-18.0105 Da) not phosphorylation (+79.9799 Da).

When analysing data from 2 – 100 peptides in the web-tool (Figure 1B), a file is used to provide the peptide sequences and their associated dta files. The Peptide + Spectra file should consist of two tab separated columns. The first column should contain the peptide sequence and potential phosphorylation site information, formatted as described here; the second column should contain the name of the corresponding spectrum file. The file can easily be created using MS Excel or another spreadsheet program and saved as a tab delimited text file. To prevent the need to upload each .dta file individually, a zip archive of the .dta files is used. To create a zip archive of the .dta files listed in the Peptide + Spectra file on a Windows based computer, there are numerous commercially available archiving programs such as the Winzip program [9] which can be used. On other operating systems such as MacOS X and Linux variants, a version of zip should be installed by default and the user should refer to the relevant documentation. Note that only zip archives can be decompressed by the server and other archive types will not work.

When run, the calculator will return the following results (Figure 2A),

• phosphosite variant: a list of phosphosite variants of the provided sequence, with the phosporylation sites considered in square brackets

• ions: the number of ions predicted from the peptide sequence

• ions matched: the number of predicted ions whose mass matched the masses in the dta file,

• p-values: the likelihood of this number of matches (defined in formula 1)

• score: phosphorylation site score (defined in formula 2).

### Efficacy of PhosCalc

To demonstrate the utility and limitations of PhosCalc, we analysed a sample of the well characterised phosphoprotein, bovine beta-casein (Sigma). A sample of bovine casein protein was digested with trypsin and analysed with an LTQ mass spectrometer. Peptides were identified using SEQUEST (ThermoFisher) on Bioworks 3.1 (full MS details, SEQUEST search parameters and peptide scores are available from the PhosCalc website). Phosphopeptides were identified from casein alpha-S1, beta-casein variant CnH and casein alpha-S2. These are products of a small related gene family and their inclusion in the preparation from Sigma was expected; this was useful as it provided a range of well characterised phosphopeptides. The observed phosphopeptides of casein αS1, αS2 and β are detailed in Supplementary Table 1. All dta files and Sequest scores can be downloaded from the PhosCalc website.

**Table 1:** The sensitivities and specificities based on the distributions of PTM score for phosphorylated and non-phosphorylated sites in known phosphopeptides.

	Sensitivity (%)	Specificity (%)	PTM score cut-off
MS2	99	82	83.97
	95	82.5	85.98
	90	82.47	86.99
			
MS3	99	39	39.9
	95	43.5	56.35
	90	48	76.95

**Figure 3:**
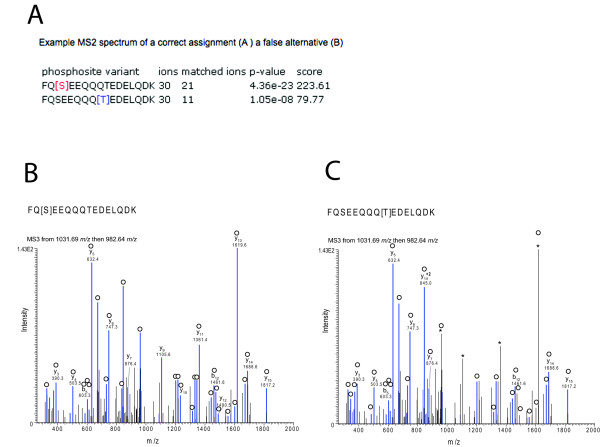
**Example of results of PhosCalc for the peptide FQSEEQQQTEDELQDK with annotated MS3 spectrum from Sequest**. A) PhosCalc output. B) Spectrum from a correct assignment. C) The same spectrum as 'B' with an incorrect assignment. Peaks marked in blue and marked with empty circles are matched to b or y ions by Sequest and * marks those peaks that do not match with the incorrect position of phosphorylation. For clarity only singly charged b and y ions without further loss of OH or NH_3 _groups are annotated, with the exception of the intense y14 2+ ion.

Peptide FQSEEQQQTEDELQDK has only two possible phosphorylation sites and they are well separated (Ser3 and Thr9). The output of PhosCalc clearly shows that the MS2 and MS3 spectra strongly support the known phosphorylation site at Ser3 (Figures 2A and 3A). The first column shows the site being assessed, the second reports how many ion peaks in the spectrum were evaluated (30) and the third column contains the predicted b or y ions matched (20 or 11 for Ser3 or Thr9, respectively). Finally the *p*-value and score are also reported. So that experienced MS users can assess how the numbers produced by PhosCalc compare with visual representations of spectra, two annotated versions of the same spectrum are provided for both the correct Ser3 assignment and the false Thr9 (Figures 2A and 3A). In this case, as the spectrum is good and the putative phosphorylation sites are well separated, several y ions (from y8 to y14; covering the portion SEEQQQT of the peptide) do not match if the phosphorylation is located on Thr9. Such matching is very clear in the MS3 spectra annotated in Figures 3B and 3C. While the differences are clear in this example it is not trivial to make these comparisons manually and PhosCalc can assist in identification of those spectra that do provide sufficient evidence to discriminate between alternative positions.

**Figure 4:**
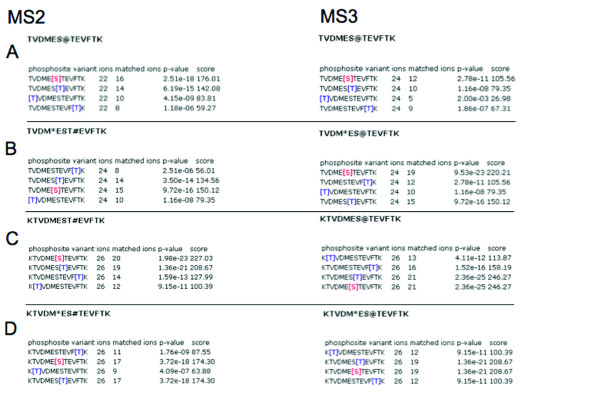
PhosCalc output for variations of KTVDMESTEVFTK.

Of all the phosphopeptides identified, KTVDMESTEVFTK provides the most detailed example because this peptide should contain only one 'true' phosphorylation site and has three alternative positions. The peptide was also observed with an oxidised methionine residue (Figures 4B and 4D) and with a mis-cleavage (K) (Figures 4A and 4C). Figure 4A shows that only with the unoxidised form of TVDMESTEVFTK can both MS2 and MS3 spectra distinguish between the correct phosphorylation site at Ser6 and Thr7. We recommend that at least two peaks should be differential between alternative positions before the phosphorylation site can be unambiguously identified. This correlates to a difference of three orders of magnitude between position scores. That it is often hard to precisely support one phosphorylation site does not demonstrate a weakness of PhosCalc but rather the limitations of the mass spectra provided. As the two main alliterative sites are next to each other, one could only expect very few ions to be discriminatory between alternative sites.

**Figure 5:**
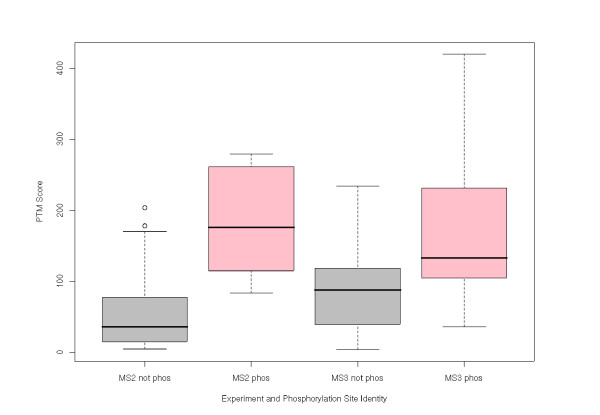
**Comparison of PTM score distributions generated by PhosCalc**. Grey boxes indicate distributions of PTM score in sites that are known not to be phosphorylated, pink boxes indicate distributions of PTM score in sites that are known to be phosphorylated.

To assess the ability of PhosCalc to distinguish between phosphorylated and non-phosphorylated sites from experimental data, we utilised the spectra of phosphopeptides from *Arabidopsis thaliana *which had been previously identified by other researchers [10-12]. All spectra were generated on an LTQ (ThermoFisher Corp.) using the conditions detailed previously. The spectra can be downloaded as dta files from the PhosCalc website. For these data an error tolerance of 0.4 Da was utilised. A total of 50 sites were analysed with PhosCalc and the PTM score of known phospho-sites was compared with those of potential sites. Figure 5 shows the PTM score distributions of each set. A clear and significant difference can be seen between the PTM score distributions of non-phosphorylated and phosphorylated sites in both MS2 (*p *< 2.51 × 10^-4^) and MS3 (*p *< 0.049), showing that the implementation is capable of distinguishing between true phosphorylated sites and non-phosphorylated sites.

We have also tested scores generated by PhosCalc with scores generated by Ascore on sample datasets and find that they are largely equivalent.

To guide the user to a useful PTM score cut-off, we calculated sensitivities and specificities based on the distributions of PTM score for phosphorylated and non-phosphorylated sites in known phosphopeptides, Table 1. The implementation of the algorithm works extremely well. We are able to obtain 99% sensitivity at a specificity of 82% in spectra from MS2 experiments, using a PTM score of 83.97 or higher. The implementation also works well with MS3 spectra, allowing a specificity of 48% at a sensitivity of 90% with a PTM score of 76.95 or higher.

Previously published studies have not used this algorithm in isolation, rather it has been used in conjunction with other measures such as MASCOT scores [2]. These pipelines assign different confidence thresholds depending on the study and type of MS. We advise that users should implement additional scoring criteria particularly regarding the sequence assignment and that PhosCalc scores and cut-offs should be chosen with care.

The PhosCalc software is a fast and simple tool for reliably identifying phosphorylation sites in mass spectrometer data. PhosCalc should find utility in laboratories carrying out phosphorylation site analyses at any scale. By using our empirical sensitivity/specificity estimations and PTM score cut-offs or those used in other studies or by comparing with PTM scores in previously curated data sets from in-house examinations, the software can be used to speed up or automate decisions on phosphorylation site identity. With low-mass accuracy data, it should be noted that when putative phosphorylation sites are close to each other on the peptide, or if the mass spectrum contains few peaks of reasonable intensity in the area of interest, there may not be enough information (from that spectrum) to discriminate between alternatives. It is important to be aware of the limitations of the spectra obtained and explicit about the levels of confidence in a particular phosphorylation site. The strength of PhosCalc is to enable users to rapidly identify those spectra which provide strong evidence for a specific phosphorylation site, even from low-mass accuracy data.

## Availability and Requirements

Project name: PhosCalc

Project home page: 

Operating system(s): Platform independent

Programming language: Perl

Other requirements: For the download version, Perl 5.6 or higher, Perl Math module, also under GPL and provided with PhosCalc download

License: GPL 3

Restrictions to use by non-academics: none

## Abbreviations

Amu: Atomic mass units; Da:= Dalton; PTM: Post-translational modification.

## Competing interests

The authors declare that they have no competing interests.

## Authors' contributions

DM developed the implementation of the algorithm and the web tool and carried out the statistical analyses, MAB assisted in making the web-tool and implemented and maintains the infrastructure upon which it runs, DJS participated in the design of the implementation and AMEJ conceived of the study, participated in its design and carried out manual curation of peptide phosphorylation site data to allow verification of the implementation.
